# A New HPV score System Predicts the Survival of Patients With Cervical Cancers

**DOI:** 10.3389/fgene.2021.747090

**Published:** 2021-12-01

**Authors:** Qunchao Hu, Yani Wang, Yuchen Zhang, Yanjun Ge, Yihua Yin, Haiyan Zhu

**Affiliations:** ^1^ Department of Radiation Oncology, Shanghai First Maternity and Infant Hospital, Tongji University School of Medicine, Shanghai, China; ^2^ Department of Gynecology, Shanghai First Maternity and Infant Hospital, Tongji University School of Medicine, Shanghai, China

**Keywords:** HPV status, multi-omics, cervical cancer, HPV score, prognosis

## Abstract

Persistent high-risk human papillomavirus (hrHPV) infection is confirmed as the major cause of cervical cancer. According to the HPV infection status, cervical cancer could be generalized as following three subgroups: HPV-negative, pure HPV-infection, and HPV-integration. Currently, the impact of HPV status on cervical cancer prognosis remains under dispute. Therefore, we explored the potential correlation between HPV status and the clinical outcome of cervical cancer, by establishing a robust prognostic predicting model based on a cervical cancer cohort using The Cancer Genome Atlas (TCGA) database. We performed an iCluster algorithm incorporating DNA copy number variation, SNP, DNA methylation, mRNA expression, and miRNA expression profile together and classified the cohort into three clusters. According to defined clusters, we established an HPV score system by weighing resultant gene alterations through random forest and COX models. This prediction tool could help to identify cervical cancer prognosis through evaluating individual HPV infection status and subsequent genetic modification, which might provide insights into HPV-related gene driven cervical cancer treatment strategies, yet its predictive power and robustness need to be further verified with independent cohorts.

## Introduction

Cervical cancer (CC) is one of the most perplexing women’s health problems and the commonest gynecological cancers, ranking fifth in the incidence rate and mortality rate of women (Bray et al., 2018). Persistent high-risk HPV (hrHPV) infection has been identified as the main event leading to cervical cancer (Cohen et al., 2019). For women, the cumulative lifetime infection rate for HPV can range from 60 to 70%, however, only a few infections persist and eventually lead to cancer (Qingqing et al., 2020). It is generally believed that the integration of the HPV genome into the host chromosome is the key genetic step in the pathogenesis of cervical carcinomas (Pett and Coleman, 2007). It is reported that the majority of HPV18 and HPV16 related cervical cancer samples were found to have structural aberrations and increased target-gene(s) expression (Cancer Genome Atlas Research Network, 2017). HPV integration usually involves the inactivation of viral E1 and E2 regions, resulting in the upregulation of oncogenes E6 and E7. Then, E6 oncoprotein degrades p53, inhibits cancer cell apoptosis and viral DNA replication. E7 is known for suppressing RB1, which abrogates cell cycle arrest and stimulates proliferation (Hu and Ma, 2018).

Previous studies have classified cervical cancer into HPV-negative cervical cancer (HPV-) and HPV-positive cervical cancer (HPV+) and reported women with hrHPV-positive cervical tumors had a substantially better prognosis than those with hrHPV-negative tumors (Lei et al., 2018). Moreover, the HPV genotype showed an independent prognostic value in early-stage cervical cancer with contradictory findings (Lai et al., 2007; Cuschieri et al., 2014). Recently, HPV-positive cervical cancer was further classified into pure HPV-infection and HPV-integration (HPV-int), according to the characteristics of HPV invasion (Wei et al., 2015; Cancer Genome Atlas Research Network et al., 2017). Whether there are differential molecular characteristics between HPV-positive cancers and HPV-negative cervical cancers remains unclear. Furthermore, whether HPV status, including HPV-negative, HPV-infection, and HPV-int, can be stably utilized as a prognostic factor remains an issue.

The current study first explored different genomic characterizations according to HPV status, including HPV-negative, pure HPV-infection, and HPV-int, using TCGA multi-omics data, then established molecular subgroups based on HPV status. Finally, we developed an HPV score model that can effectively predict the survival of cervical cancer.

## Materials and Methods

### Data Collection

Genomic, transcriptomic, and epigenomic data for cervical squamous cell carcinoma and endocervical adenocarcinoma (CESC) were downloaded from the TCGA database. We downloaded MAF files for cervical cancer (the reference genome of the MAF file is HG19) using the R-package of TCGAbiolinks, which contains the mutation detection results from 297 samples, while SNP Copy Number segment data for 287 cervical cancer samples and the methylation microarray data for 299 cervical cancer samples were drawn from Fire Browse (http://firebrowse.org/). The mRNA expression profiles of 307 cervical cancer samples were downloaded with UCSC Xena (https://xenabrowser.net/datapages/). There were 284 samples with multi-omics data, including DNA mutation, copy number alteration, methylation, mRNA, and miRNA expression profiles. Clinical data were downloaded from the TCGA data portal, including primary tumor histological type and grade, HPV infectious status, and FIGO stage. The follow-up analysis was based on 283 samples with the full information collected.

### Data Analysis

#### The Differentially Expressed Genes and Differentially Methylation Genes in Cervical Squamous Cell Carcinoma and Endocervical Adenocarcinoma From The Cancer Genome Atlas

The differentially expressed genes (DEGs) and methylation genes (DMGs) between the HPV + group and the HPV- group were identified by R programming, using the limma package and the ChAMP package, respectively. As for DEGs of mRNA and miRNA, fold change values larger than 1.5 and *p*-value less than 0.05 were set as the cut-off criteria. DMGs were predicted based on the β-value file of CpG site probes, Δβ, and *p*-value. Only regions with |Δβ| larger than 0.1 and *p*-value less than 0.05 were identified as DMRs.

#### Gene Set Variation Analysis

Initially, we divided cervical cancer samples into two groups as HPV infected (HPV-positive) and non-HPV infected (HPV-negative), then downloaded hallmark reference gene set (c2. cp.kegg.v7.1) from the MSigDB database (https://www.gsea-msigdb.org/gsea/index.jsp) and set the *p*-value less than 0.05 and *t*-test value over 2 as cut-off criteria. We performed the GSVA enrichment analysis between two defined groups using R-package of GSVA (version 1.20.0), and also assessed the relative enrichment of gene sets across defined groups using a non-parametric approach, estimating changes in the activity of pathways and biological processes. Patients from the CESC cohort were divided into two groups according to the HPV infectious status. Genomic events like somatic mutations and somatic copy number alterations (CNAs) of samples from different groups were analyzed with the MutSigCV and GISTIC analysis modules in GenePattern (https://cloud.genepattern.org) accordingly. The mutation landscape was visualized by using the R-package maptools.

#### Subgroup Identification and Analysis of Different Genes Expression

As for subgroup identification, we integrated various molecular platforms by using iCluster, including DNA copy number, SNP, DNA methylation, mRNA expression, and miRNA expression profile (Keck et al., 2015). Then, the differentially expressed genes between subgroups of cervical cancer patients were screened by using the limma package in R. The screening criteria were |logFC| >log2 (1.5) and *p*-value < 0.05.

#### Assessment of Infiltrated the Immune Cell

The “Cell type Identification By Estimating Relative Subsets Of RNA Transcripts (CIBERSORT)” algorithm (https://cibersort.stanford.edu/) was performed to complete the calculation, and each sample was assigned with a *p*-value. Samples with CIBERSORT output *p*-value less than 0.05 were gathered for subsequent analysis. Then it was used to indicate the composition of the tumor infiltrated immune cells with LM22 signature as a reference, which is based on the gene expression profiling of complex tissue (Newman et al., 2015). The proportion of 22 subtypes of immune cells was obtained, including myeloid subgroup, NK cell, plasma cell, juvenile, memory type B cells, and seven subgroups of T cells. Pearson’s Chi-squared test was launched to estimate different immune cell infiltration statuses and the visualization was presented in the R program.

#### HPV Score Model Construction

According to the different expression gene patterns of the three clustered subgroups (as described previously in the section on “Subgroup identification and analysis of different genes expression”), the random forest method was applied to filtrate the redundant genes. Then, multivariate Cox regression combing patients’ survival data was used to correlate candidate genes and divide them into positive or negative regulation groups. We took a *p*-value less than 0.05 as a threshold. Ultimately, the score of GCI was used to estimate the HPV score model as follows (Sotiriou et al., 2006; Zeng et al., 2019; Zhang et al., 2020).

HPVscore = scale(∑X−∑Y)

In this formulation, X indicates the expression value of the gene set with positive co-efficient(s), while Y refers to the negative correlation in Cox regression, and scale represents the standardization process.

The optimal threshold of predictive HPV score was determined by surv_cutpoint algorithm of survival R package. This function is used to explore the optimal cutoff value for one or multiple continuous variables by using the maximally selected rank statistics from the “maxstat” R package. Thus, the cervical cancer patients were divided into HPV high score and HPV low score groups according to individual calculation with the formula above. The survival difference between the HPV high score and low score was determined by the log-rank test with a *p*-value less than 0.05 as statistically significant.

### Statistical Analysis

**FIGURE1:**
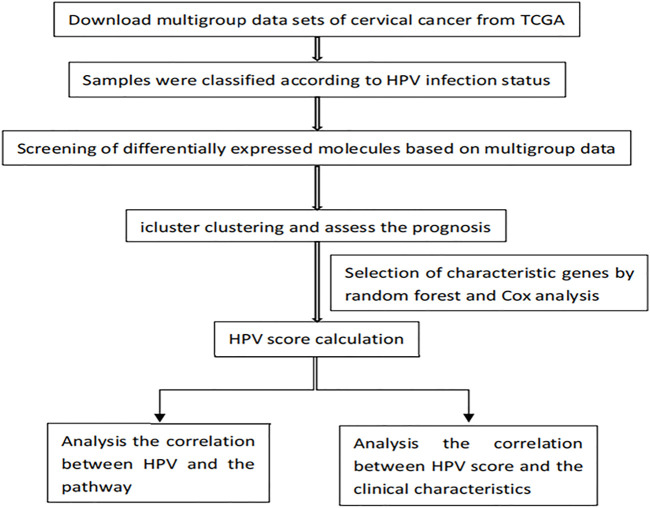
Flow chart.

R software (version 3.6.2; https://www.r-project.org/) was used in data mining and statistical analyses. Wilcoxon test was used to evaluate the differences between the defined groups. The Student t-test, Pearson’s Chi-squared test, or χ^2^ test was applied to distinguish different expression profiles regards to defined groups. The survival curves were determined by the Kaplan-Meier method, while the log-rank test compared the clinical outcome differences. Based on specified genes of identified subgroups, the predictive HPV score model was constructed. Receiver operating characteristic (ROC) and area under the curve (AUC) were drawn with ROC and timeROC package in the R program to evaluate the model predictive ability and robustness. A *p*-value of <0.05 was considered statistically significant. The flow chart is shown in Figure 1.

## Results

### Multi-Omics Data Analysis According to HPV Infection Status

**FIGURE2:**
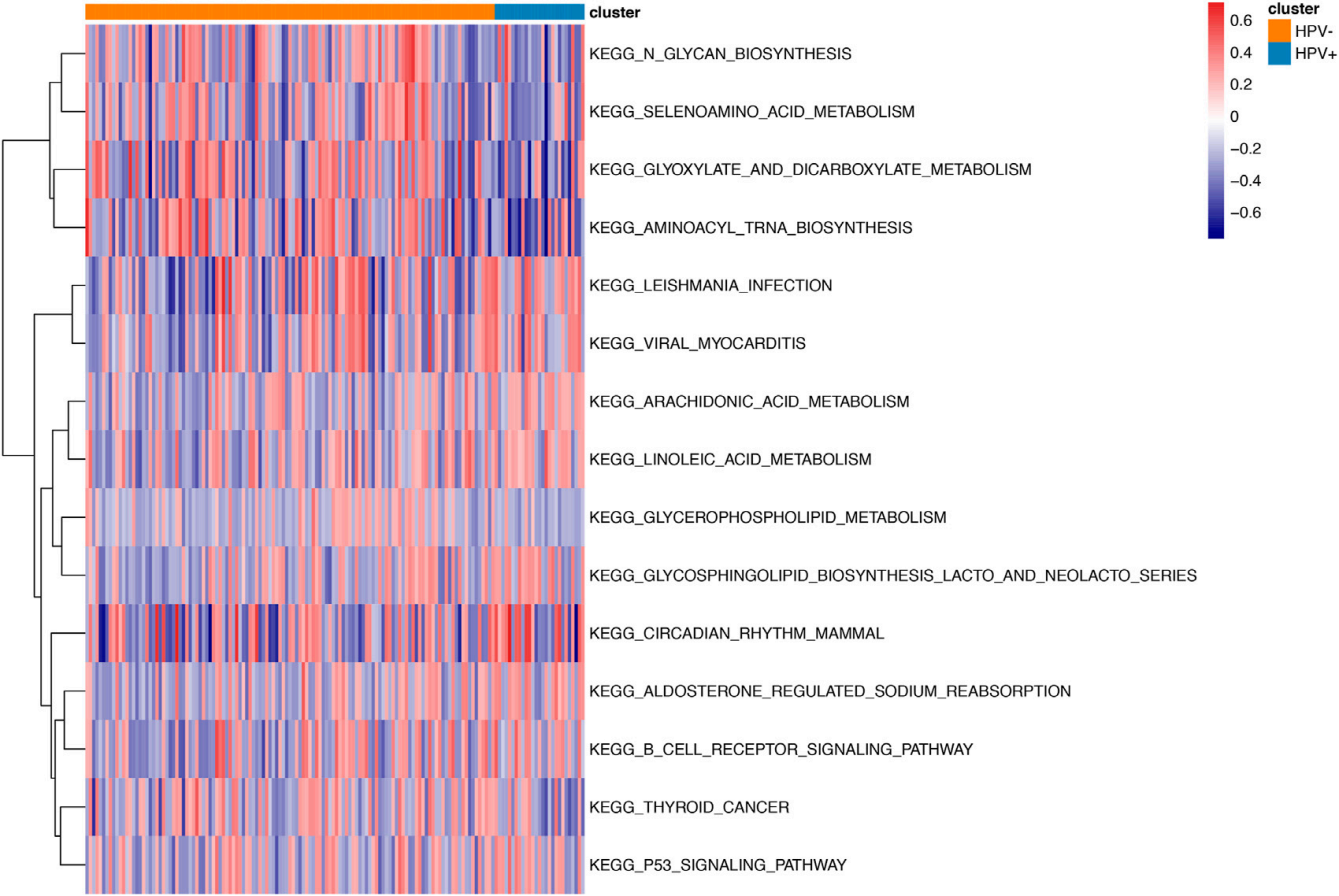
GSVA enrichment analysis between HPV positive and negative samples. Different colors stand for different status of biological pathway: red means activation and blue means inhibition.

We identified 263 differentially expressed mRNA, 28 differentially expressed miRNA, and 73 differentially DNA methylation sites between HPV-negative and HPV-positive groups. As shown in Figure 2, Gene Ontology clustering (GO) and Kyoto Encyclopedia of Genes and Genomes (KEGG) pathway analysis indicated that non-HPV related cervical cancers were associated with enrichment of biological function such as glycan biosynthesis, selenoamino acid metabolism, glyoxylate, and dicarboxylate metabolism, as well as aminoacyl Trina biosynthesis, when compared to those patients with HPV positive.

**FIGURE 3:**
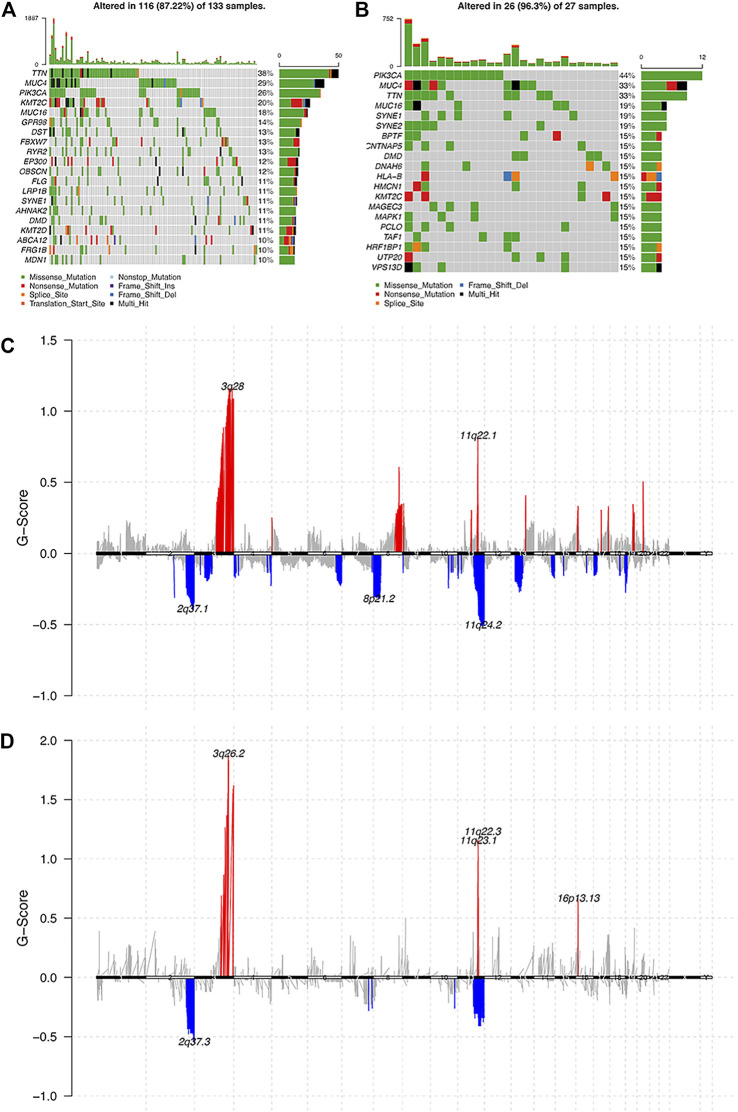
Analysis of the molecular characters between pure HPV-infection and HPV-int samples. **(A)**: Gene mutation in pure HPV-infection samples, **(B)**: Gene mutation in HPV-int samples, **(C)**: Distribution of CNV regions among pure HPV-infection samples, **(D)**: Distribution of CNV regions among HPV-int samples.

As for genomic aberrations between cervical cancer with pure HPV-infection and HPV-int, we found 98 genes SNP variants of significant differences, such as CNTNAP5, MYO7B, LMOD3, etc. Compared to pure HPV-infection, the HPV-int group presented a higher frequency of CNV events, including copy number gain in 1933 genes and copy number loss in 187 genes. As shown by the gene mutation in Figure 3AB, the most common mutation sites in pure HPV-infection and HPV-int samples were located in the genes TTN, MUC4, PIK3CA, and MUC16. HPV-int group demonstrated a higher frequency of gene alteration than the non-integrated group (96.3 vs 87.2%). PIK3CA gene missense mutation was found in 12 cases (44%, 12/27, Figure 3B). Figure 3CD shows the copy number variation enrichment in different genomic regions between pure HPV-infection and the HPV-int samples set. Copy number amplification is mainly located in chromosome 3q28 in the HPV-infection group and chromosome 3q26.2 in HPV-int set, while the copy number loss was mostly located in 2q37.1 in the HPV-infection samples and 2q37.3 in the HPV-int set.

### HPV Related Subgroup Classification and Signature Analysis

**FIGURE 4:**
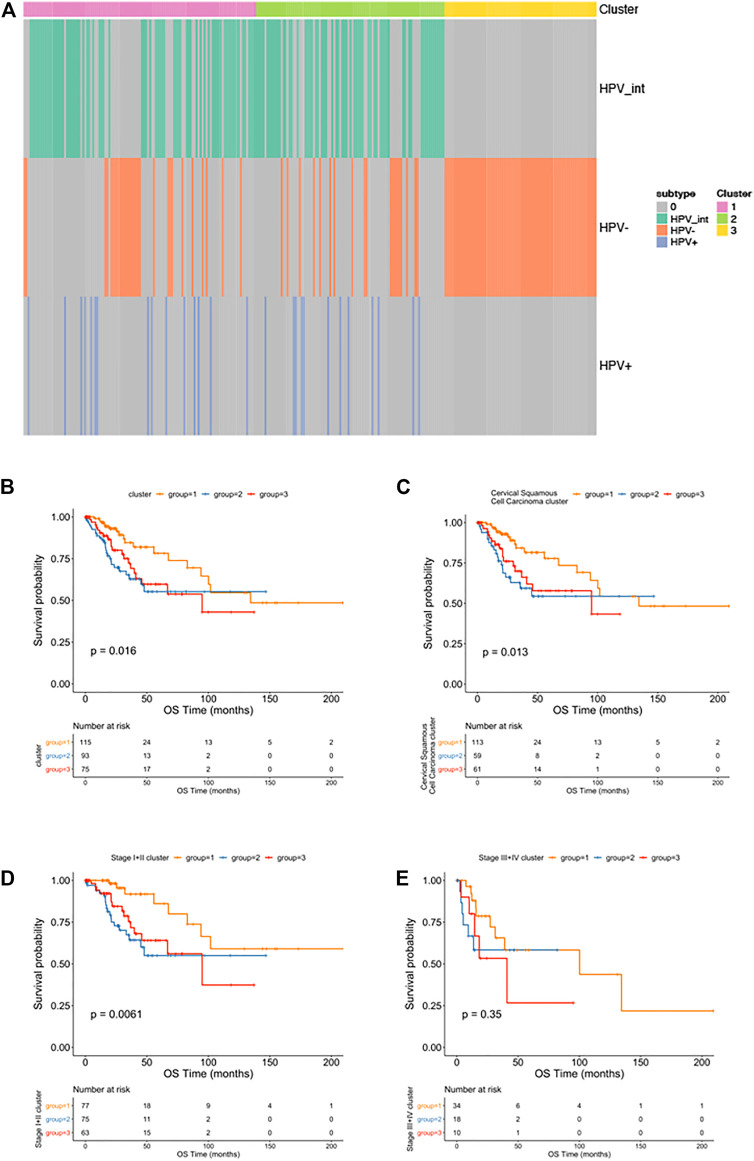
Multi-omics clustering analysis. **(A)**: The cluster-of-clusters analysis separated cervical cancers into three subgroups; **(B–D)**: Kaplan-Meier analysis of overall survival stratified by three subgroups among all cervical cancer patients **(B)**; among squamous cervical cancer patients **(C)**; among patients with stage I-II cervical cancers **(D)** and stage Ⅲ-Ⅳ cervical cancers **(E)**.

We then performed an iCluster algorithm, incorporating five different omics of DNA copy, SNP, DNA methylation, mRNA, and miRNA expression profiles together and sorted them into three HPV-related clusters. As shown in Figure 4A, while cluster 1 and cluster 2 are mainly pure HPV-infection and HPV-int, cluster 3 is of a great proportion of non-HPV-related cervical cancer patients. Patients of cluster 1 presented superior overall survival to that of cluster 2 and cluster 3, though there is no statistical difference between cluster 2 and cluster 3, especially in the squamous cell cancer patients (Figures 4B,C). In addition, cluster 1 showed an improved OS compared to cluster 2/3 among patients in stage I/II cervical cancer (*p* = 0.0061). Among those three clusters, only patients of cluster 3 reached the median overall survival of 94.8 months (95%CI: 45.3–144.4). However, there was no significant difference in OS among the three clusters in patients with stage III/IV (Figures 4D,E).

**FIGURE 5:**
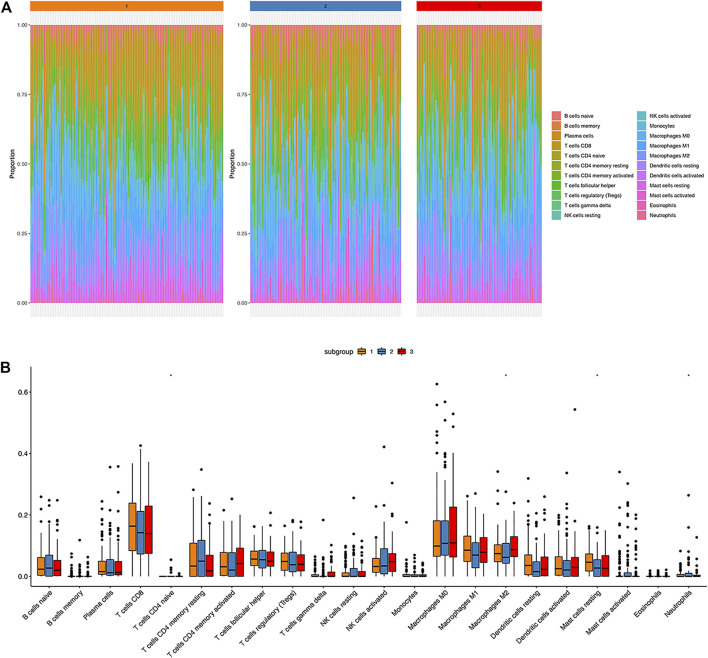
Immune landscape atlas in three HPV clusters. **(A)**: The distribution of 22 types of immune cells in three classified clusters; **(B)**: Percentage distribution of immune cells in three classified clusters; ns: *p* > 0.05, *: *p* ≤ 0.05, **: *p* ≤ 0.01, ***: *p* ≤ 0.001, ****: *p* ≤ 0.0001.

CIBERSORT method combined with LM22 characteristic matrix was used to further estimate the differences of infiltrating immune cell subsets among those three clusters. The proportion of immune cells in each sample varied within and between groups (Figure 5A). There were significant differences in subsets of resting mast cells, macrophage M2, Neutrophils, and CD4 naïve T cells among the three groups (Figure 5B).

**FIGURE 6:**
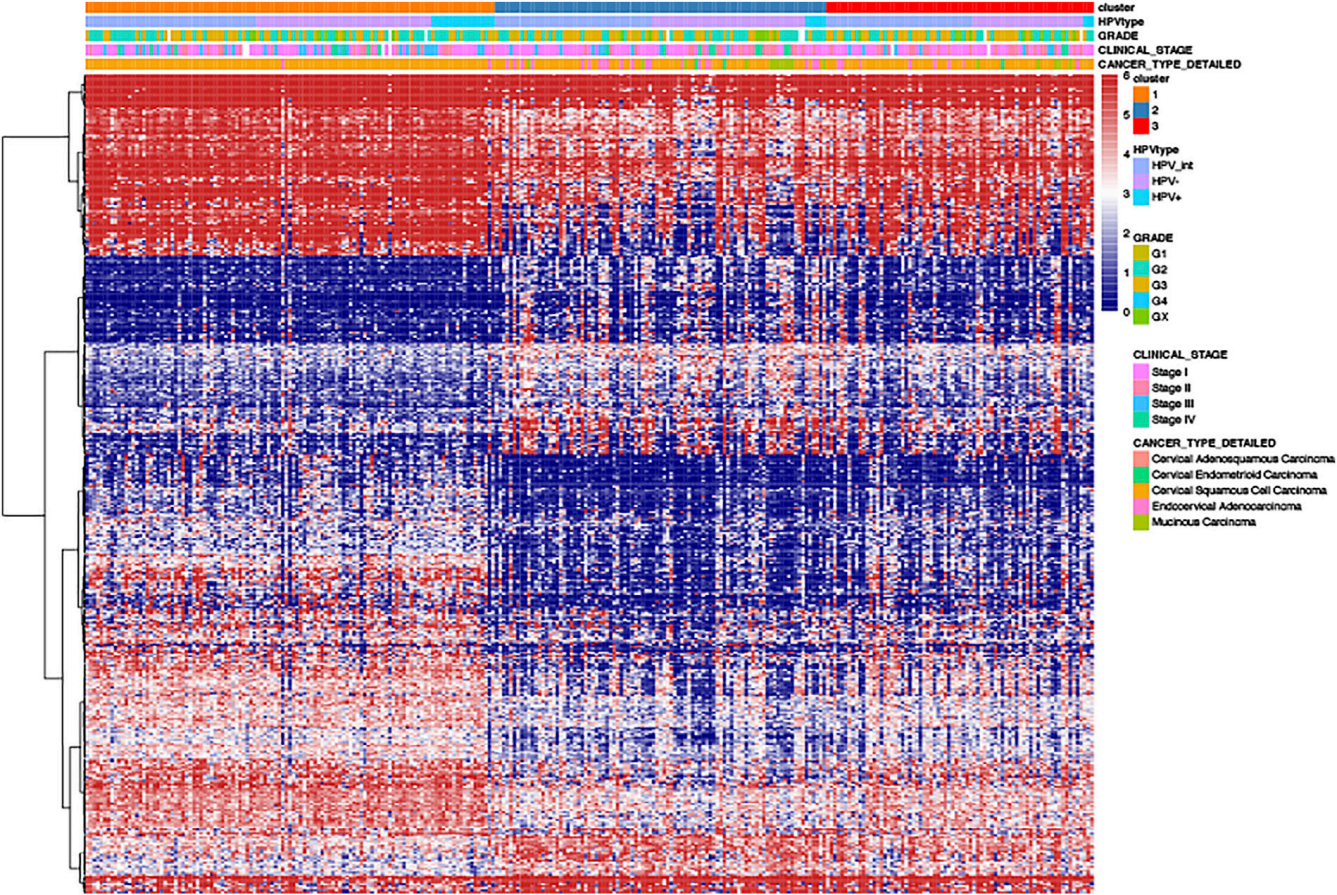
The heat-map of differential gene distribution in cervical cancer stratified by HPV related clusters, HPV status, grades, stages, pathological types.

We analyzed the differential genes among these three clusters using the R-package limma. Finally, 460 differential expressed genes were identified (Figure 6).

### HPV Score Model Construction

**FIGURE 7:**
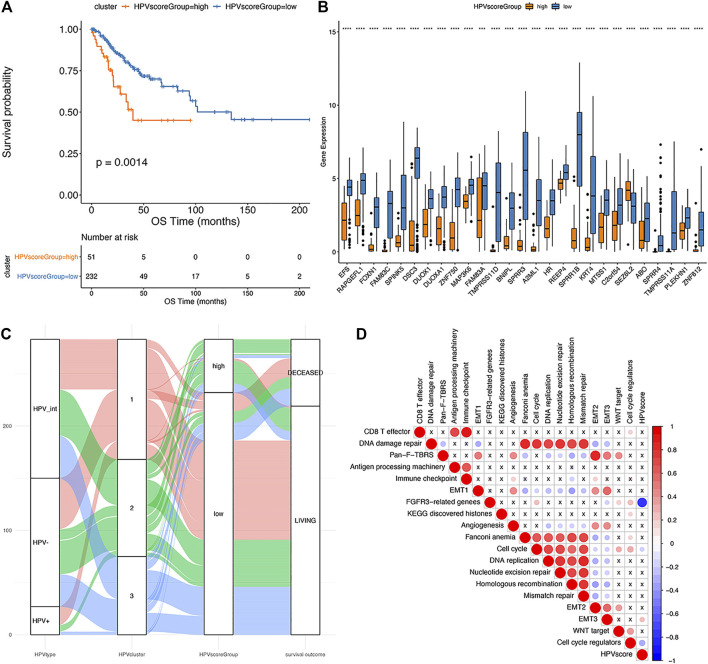
The establishment of HPV score system. **(A)**: Kaplan-Meier analysis of overall survival in cervical cancer patients stratified by HPV score; **(B)**: The expression of HPV score associated genes in different HPV score groups (significance analysis by *t*-test); **(C)**: The alluvial plot showing the correlation among HPV status, iCluster clustering and HPV score; **(D)**: Pearson analysis of HPV score and its associated genes (The negative correlation is marked in blue and positively correlated with red. The X in the graph indicates that the correlation is not significant, and the larger the circle is, the more significant the correlation is).

Since it is known that HPV status alone (HPV-negative, pure HPV-infection, HPV-int) cannot effectively predict the prognosis of cervical cancer, this study aimed to develop a multi-factor model, called the “HPV-score,” according to virus infection status and resultant gene alterations according to defined clusters. We selected the characteristic genes that are most relevant to classification among those 460 differential genes using a random forest algorithm. The optimal threshold point (cutoff = 1.15482) for the classification of HPV-score was determined according to the surv_cutpoint function in the R-package survminer, and the samples were divided into two categories: HPV-score high and HPV-score low. As shown in Figure 7A, the group with HPV-score high had a relatively poorer prognosis compared to those with low HPV-score. The HPV-score-related genes identified are shown in Figure 7B. The correlation between HPV status, HPV-related clusters, and HPV-score are was depicted in Figure 7C. HPV-score was considered negatively correlated with genes from the FGFR3 pathway and cell cycle regulation and positively correlated with epithelial-mesenchymal transition (EMT) biological functions, which might promote cancer cell proliferation, invasion, and migration (Figure 7D).

**FIGURE 8:**
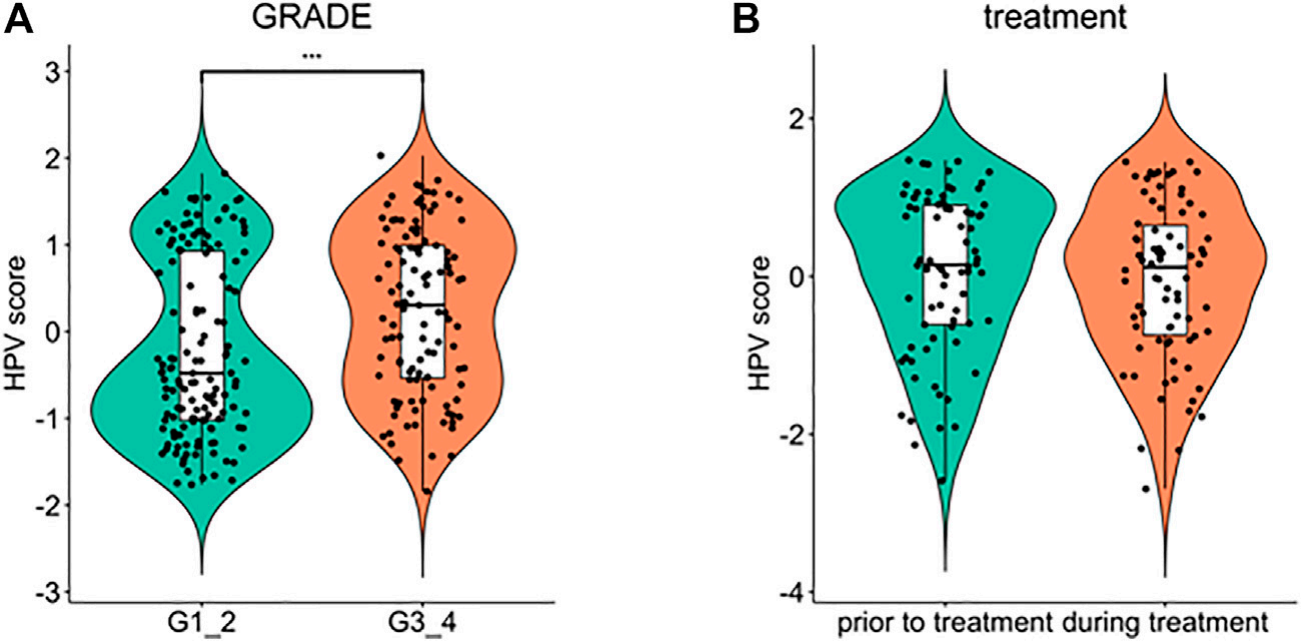
Comparison of HPV score variation according to cancer cell differentiation **(A)** and treatment time schedule **(B)**.

**FIGURE 9:**
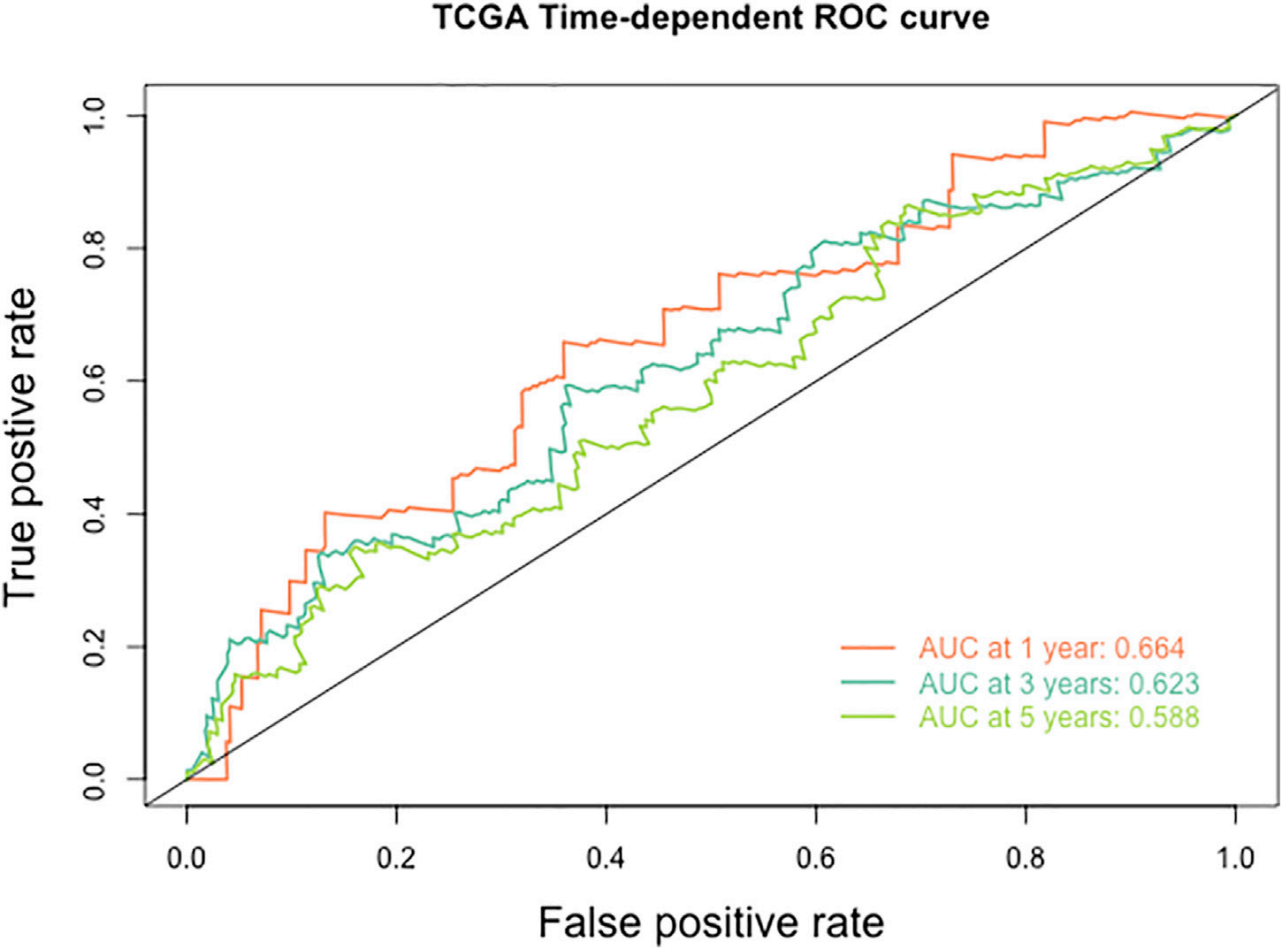
The ROC curve of HPV score for predicting the survival rate of patients with cervical cancer in TCGA database.

Furthermore, we explored the relationship between HPV-scores and histological differentiation and found poor differentiation (grade3-4) showed higher HPV-scores than well differentiation (grade1-2) (Figure 8A). The HPV-scores showed no change before and after treatment (Figure 8B). Finally, we used the HPV-score to predict the 1,3,5-years survival rate among the TCGA CESC dataset, and the AUC values presented as 0.664, 0.623, 0.588 accordingly (Figure 9).

## Discussion

Cervical cancer is one of the most common gynecological cancers (Bray et al., 2018). Although it is generally believed that persistent infection of high-risk HPV is the main etiology of cervical cancer (Cohen et al., 2019), a variable proportion of tumors are reported to be negative for hrHPV (Lei et al., 2018). A previous study reported that women with hrHPV-positive cervical tumors had a substantially better prognosis than women with hrHPV-negative tumors (Lei et al., 2018). However, whether the pathogenesis of these two types of cervical cancer is different is unclear. In the current study, we firstly explored the different molecular characteristics between HPV-positive and HPV-negative cervical cancer and revealed that HPV-positive tumors were depleted in glycan biosynthesis and metabolism. Interestingly, in a recent study, Yang et al. explored the compositional and functional alterations on vaginal samples between HPV16-positive and age-matched HPV-negative controls, and demonstrated that HPV16-positive candidates were enriched in metabolism and membrane transport, and depleted by glycan biosynthesis and metabolism, and replication and repair, when comparing to the HPV-negative samples (Yang et al., 2020). These results were consistent with our results, suggesting metabolic reprogramming involved in HPV-induced cervical cancer.

It is generally believed that the integration of the HPV genome into the host chromosome is the key genetic step in the pathogenesis of cervical carcinomas (Pett and Coleman, 2007). We then explored the genetic differences between pure HPV-infection and HPV-int tumors and verified that PIK3CA, TTN, MUC4, KMT2C, and SYNE1 were common somatic mutations both in pure HPV-infection and HPV-int women, suggesting that these genes play an important role in HPV-associated cervical cancer. Along similar lines, PIK3CA is reported as the most frequently mutated oncogene in cervical cancer associated with HPV infection, and PIK3CA mutation is the only variable significantly associated with disease recurrence (Kannan et al., 2017; Beaty et al., 2020). In addition, the copy number amplification of pure HPV-infection and HPV-int samples was 3q28 and 3q26.2, and the deletion sites were 2q37.1 and 2q37.3, respectively, which was consistent with our previous report (Xu et al., 2021). Copy number alteration driven dysregulated genes, such as PI3KCA, PI3KCB, DVL3, WWTR1, and ERBB2, are believed to play an important role in regulating immune cell infiltration, which might help to predict the prognosis of cervical cancer (Wen et al., 2019).

We then explored molecular classification based on HPV status. Samples were classified by iCluster clustering, and three subgroups were obtained. Cluster 1 and Cluster 2 are characterized as HPV-positive, while Cluster 3 is characterized by being HPV-negative. Interestingly, when we compared the prognosis among the three subgroups, we detected both Cluster 2 and 3 showed worse overall survival as compared to Cluster 1. Previous studies have shown that women with hrHPV-positive cervical tumors had a substantially better prognosis than women with hrHPV-negative tumors (Lei et al., 2018), which is consistent with our results, the majority of pure HPV-infection, as well as HPV-int patients (Cluster 1), showed a better prognosis than HPV-negative women (Cluster 3). However, there were a proportion of HPV-positive tumors (Cluster 2) that showed a similarly poor prognosis as compared with HPV-negative women (Cluster 3). Therefore, simply based on the fact that HPV negative, pure HPV infection, and HPV integration cannot clearly predict the prognosis of cervical cancer.

To identify the genetic heterogeneity among the three subgroups and determine their relationship with the prognosis of the disease, an HPV-score system was established for all the samples. Patients with high HPV-scores showed a much worse prognosis than those with low HPV scores, suggesting that the calculation based on HPV score is a good prognostic predictor. Moreover, a high HPV-score was considered to be correlated with poor cancer cell differentiation, which might suggest that tumors with high HPV scores present more aggressive biological behavior. Additionally, this system was relatively stable, and not subject to treatment changes, as it showed no difference before and after treatment within a chosen cohort.

This HPV-score showed a significantly positive correlation with EMT3 biological functions, while negatively related to FGFR3-pathway genes and cell cycle regulation. Presumably, FGFR3 kinase plays a key role in regulating infected epithelium cells by limiting HPV replication, inhibiting tumorigenesis, and tumor growth (Bersani et al., 2017).

To further explore the immune basis of different HPV statuses, we investigated the correlation between HPV status and infiltrating immune cell subsets. The proportion of immune cells in each sample varied within and between groups. Notably, significant differences existed in the composition of resting mast cells, macrophage M2, Neutrophils, and CD4 naïve T cells among the three groups. A high fraction of activated mast cells was considered as independent factor associated with adverse outcomes in cervical cancer (Wang et al., 2019; Yang et al., 2019). Mast cells may contribute to an immunosuppressive environment that enables the persistence of HPV E7 protein induced pre-cancerous lesions (Bergot et al., 2014). In an esophageal squamous cell carcinoma model, HPV16 infection promoted an M2 macrophage phenotype, thus, enhanced the invasion and metastasis (Yuan et al., 2021a). Tumor-associated neutrophils (TAN) can promote the formation of the tumor microenvironment. Besides, it is believed that a high proportion of neutrophil infiltration facilitates microenvironment formation for tumor progression and eventually results in a poorer prognosis in solid tumors. CD4^+^ T cells play a crucial role in eliminating the virus. After activation, naive CD4^+^ T could enhance cellular or humoral immune response through activating T-helper 1 (Th1) and Th2 subsets, respectively (Yuan et al., 2021b). Collectively, the immune medium constitutes a crucial role in regulating HPV-related cancers.

The main strength of our study is that we established an HPV scoring model that effectively predicts the prognosis of patients with cervical cancer. The main limitations of our study are that we only used the TCGA database. Another limitation is connected to the fact that the majority of the samples were pathological types of squamous cell carcinoma, meaning there was a lack of other pathological types and HPV infection status of the results. The status of HPV is mainly detected before treatment, and the lack of longitudinal data during treatment and follow-up may affect the extrapolation of the HPV-score during treatment and follow-up.

In conclusion, we explored the potential correlation between HPV status and clinical outcome of cervical cancer and then established a HPV-score that can effectively predict the prognosis of patients with cervical cancer. This scoring system demonstrated good consistency with clinicopathologic characteristics and may provide guidance for HPV-related cervical cancer treatment decision-making in the future.

## Data Availability

The original contributions presented in the study are included in the article/Supplementary Material, further inquiries can be directed to the corresponding authors.
